# What’s inside matters: an assessment of the family planning content of digital self-care platforms

**DOI:** 10.1186/s12978-024-01848-4

**Published:** 2024-07-30

**Authors:** Sarah Brittingham, Lauren Mitchell, Trinity Zan

**Affiliations:** FHI 360, 359 Blackwell Street, Suite 200, Durham, NC 27701 USA

**Keywords:** mHealth, eHealth, Digital health, Contraception, Family planning, Digital self-care

## Abstract

**Background:**

Digital technology has proliferated rapidly in low- and middle-income countries in recent decades. This trend will likely persist as costs decrease, dramatically expanding access to reproductive health and family planning (FP) information. As many digital tools aim to support informed choice among individuals with unmet contraceptive need, it is essential that high-quality information is provided. We set out to assess the accuracy and comprehensiveness of FP content in select user-facing digital self-care platforms.

**Methods:**

We identified 29 digital tools in circulation between 2018–2021 and selected 11 that met our eligibility criteria for analysis. Referencing global guidance documents such as the Family Planning Handbook, Medical Eligibility Criteria for Contraceptive Use, and the Digital Health for Social and Behavior Change High Impact Practice Brief, we developed an original rubric outlining 12 key content areas necessary to support informed, person-centered counseling. We applied this to each tool, enabling assignment of a numerical score that represents content accuracy and comprehensiveness across the 12 key areas.

**Results:**

FP content of digital tools varied greatly in accuracy and comprehensiveness. Of the 12 identified key content areas, 5 were included in all 11 tools, while 6 were addressed inconsistently or not at all. Four content areas were the most accurate and comprehensive: complete list of modern methods, duration of protection, dual method use, and return to fertility. The lowest scoring content areas were side effect management, non-contraceptive benefits, effectiveness, side effects, and instructions for use.

**Conclusions:**

Complete, accurate, and evidence-based FP content is a foundational element of quality digital self-care. Inaccuracies and omissions can impact individual user experiences and decision-making in critical ways. FP content quality should be verified before digital tools are scaled or researched at the programmatic level. From this exercise, we developed a checklist for use in conjunction with global guidance documents to improve future FP content of user-facing digital tools.

**Supplementary Information:**

The online version contains supplementary material available at 10.1186/s12978-024-01848-4.

## Abstract (plain language summary)

Digital tools are increasingly used to reach people in low- and middle-income countries with reproductive health information and links to services. We set out to understand the quality of the information available in digital tools relating to family planning. To do this, we searched for a set of tools and applied eligibility criteria, ultimately identifying 11 tools for assessment. To assess their content, we developed a rubric based on cornerstone documents in family planning. This rubric contains 12 FP content areas (such as method effectiveness, duration of protection, etc.) and was applied to assess accuracy and comprehensiveness of all FP content. The FP content of digital tools varied greatly in accuracy and comprehensiveness. Of the 12 key content areas we identified, 5 were included in all 11 tools; 6 were addressed inconsistently or not at all. Four content areas were the most accurate and comprehensive: complete list of modern methods, duration of protection, dual method use, and return to fertility. The lowest scoring content areas were side effect management, non-contraceptive benefits, effectiveness, side effects, and instructions for use. Digital tool users have the right to accurate, comprehensive FP information, and gaps in quality can impact users of digital tools in various ways. Quality of FP content should be checked and verified before digital tools are scaled or resources are used to research them. Based on this exercise, we developed a checklist for use in conjunction with global guidance documents to improve the FP content of user-facing digital tools.

**Supplementary Information:**

The online version contains supplementary material available at 10.1186/s12978-024-01848-4.

## Résumé

**Contexte:**

La technologie numérique a proliféré rapidement dans les pays à revenu faible et intermédiaire au cours des dernières décennies, une tendance qui devrait se poursuivre à mesure que les coûts diminuent, élargissant considérablement l’accès aux informations sur la santé reproductive, y compris la planification familiale (PF). Étant donné que de nombreux outils numériques permettent de faire des choix éclairés, il est essentiel que les informations fournies soient de grande qualité. Nous avons entrepris d’évaluer l’exactitude et l’exhaustivité du contenu sur la PF dans certains outils numériques destinés aux utilisateurs.

**Méthodes:**

Nous avons identifié 29 outils numériques utilisés entre 2018–2021 et en avons sélectionné 11 pour l’analyse en fonction de nos critères d’éligibilité. Nous avons élaboré une rubrique basée sur des documents d’orientation mondiaux, notamment le Manuel de planification familiale, les Critères d’admissibilité médicale pour l’utilisation de contraceptifs et l’aperçu du pratique a haut impact Sante Numérique pour un changement social et comportemental, reflétant 12 domaines de contenu clés du conseil éclairé et centré sur la personne. Nous avons appliqué cette méthode à chacun des outils, ce qui nous a permis d’attribuer une note quantitatif représentant l’exactitude et l’exhaustivité du contenu de chaque outil en matière de PF dans les 12 domaines de contenu.

**Résultats:**

Le contenu des outils numériques en matière de planification familiale varie considérablement en termes de précision et d’exhaustivité. Sur les 12 domaines clés que nous avons identifiés, 5 ont été inclus dans les 11 outils; 6 domaines ont été traités de manière incohérente ou pas du tout. Quatre domaines ont été les plus précis et les plus complets: la liste complète des méthodes modernes, la durée de la protection, l’utilisation d’une double méthode et le retour à la fertilité. Les domaines les moins bien notés étaient la gestion des effets secondaires, les avantages non contraceptifs, l’efficacité, les effets secondaires et le mode d’emploi.

**Conclusions:**

Un contenu complet, précis et fondé sur des données probantes est un élément fondamental de la qualité des soins. Les inexactitudes et les omissions peuvent avoir un impact critique sur l’expérience et la prise de décision des utilisateurs. La qualité du contenu de la PF doit être contrôlée et vérifiée avant que les outils numériques ne soient mis à l’échelle ou que des ressources ne soient utilisées pour leur recherche. Sur la base de cet exercice, nous avons élaboré une liste de contrôle à utiliser en conjonction avec les documents d’orientation mondiaux pour améliorer le contenu PF des outils numériques destinés aux utilisateurs.

## Background

As access to digital technology continues to expand across the globe, evidence is mounting that digital tools can increase knowledge about family planning and reproductive health (FP/RH), facilitate informed decision-making processes and access to FP methods, and enable individuals to self-screen to promote and protect their reproductive health—all important components of self-care [1–4]. WHO’s Guideline on Self-Care Interventions for Health and Well-Being recognizes that digital health can provide “accurate and tailored information on specific healthcare interventions and technologies” [1]. The number of digital health interventions that provide access to FP/RH information and care to various population groups in low- and middle-income countries has grown tremendously over the past decade. With the addition of artificial intelligence-driven tools such as ChatGPT, it is likely that these interventions will expand at an even faster pace.

Just as access to high quality contraceptive services is a human right, so is access to high-quality information about contraception. However, to our knowledge, there is currently no resource that assesses the content quality of user-facing digital FP tools. Our primary objective was to systematically assess the content of digital FP tools for comprehensiveness and accuracy across 12 content areas reflecting core elements of counseling for informed choice, as well as to provide actionable insights to the strengthen this content. Following this analysis, we recommended a list of vetted digital FP tools for adaptation and/or scale-up and created a checklist that can be applied to assess and improve digital FP content quality.

## Methods

### Tool landscaping

To source user-facing digital tools for family planning, we implemented a purposive sampling approach. We reviewed existing compendiums and resources, performed web searches, and conducted targeted outreach with family planning collaborators (Table 1). Through this process, we compiled a list of 29 tools. To be included in the analysis, tools were required to meet the following eligibility criteria:

- Contain detailed FP content with the (stated or implied) aim of increasing individuals’ knowledge about FP
- Designed for and deployed to individual users in low- and middle- income countries
- Content available in English or French
- Include fixed or static content (e.g., content on a website or in an app that does not change as opposed to a social media campaign with short-lived and rotating messages or other dynamic, user-generated, or social-media based content)
- Is in use at time of search (2020–2021) or use since 2017
- Delivered via a widely accessible platform such as SMS, chatbot, smartphone application, or website
- Tool owner was willing to provide access to content in a format that facilitated review, such as (but not limited to) MS Word or MS Excel

**Table 1:** Digital tool sources

Source Type	Sources Reviewed
Compendiums	• Digital Health Atlas • ORB Library • Digital Health Compendium​ • mHealth Compendium
FP/RH resources	• Global Goods Guidebook • High Impact Practice briefs: o Digital Health for Social and Behavior Change: New technologies, new ways to reach people o Digital Health to Support Family Planning Providers: Improving knowledge, capacity, and service quality • Global Health eLearning Center • Peer-reviewed articles
Websites	• Maternal and Child Survival Program • K4Health
Targeted Outreach	• Tech4Youth Initiative (UNFPA) • PSI • John Snow, Inc.

### Development of rubric to assess content quality

Based on review of global guidance documents such as the High Impact Practice Briefs, the Family Planning Handbook, and Medical Eligibility Criteria for Contraceptive Use, we decided to evaluate the content of user-facing tools for family planning across 12 key content areas (Table 2) that reflect essential elements of informed choice counseling. For the criteria that tools should cite all modern methods, we adopted the widely used categorization proposed by Hubacher and Trussell, though we acknowledge that these categories do not necessarily align those of contraceptive users [5, 6]. We consulted with our medical advisor—who has clinical FP knowledge and experience contributing to the development of key content resources for FP–to assign each key content area a weighted value based on relative importance for informed choice counseling, with a weighted value of 1.0 representing the highest importance (see Table 2). We incorporated the key content areas, as well as general information about the tool (i.e., level of operation (global, country, etc.), objectives, targeted population, evidence of effectiveness) into an original rubric for content review and analysis. Our medical advisor tested, iterated, and tailored the rubric prior to initiating analysis.

**Table 2:** Content area assessment criteria and assigned weights

Content area	Assessment criteria	Weight
Reproductive intentions	Ask or acknowledge relevance of how long user would like to prevent pregnancy	.6
Complete list of modern methods	Provide a complete list of modern FP methods available in the contexts where tool was deployed	1
Duration of protection	Contain comprehensive and accurate information about duration of protection afforded by each FP method	1
Dual method use	Inform that only condoms offer protection against HIV and sexually transmitted infections and encourage their use in combination with other family planning methods	1
Return to fertility	Describe impact on return to fertility of injectables and lack thereof for other FP method	1
Discreetness	Include accurate information on which FP methods can be used without a partner or parent’s knowledge	.7
Mechanism of action	Provide accurate description of the mechanism of action of each family planning method	.6
Side effects	Include accurate and complete information about common side effects by FP method	1
Instructions for use	Accurate inclusion of instructions by method	1
Effectiveness	Inclusion of accurate typical use effectiveness by FP method	1
Non-contraceptive benefits	Inclusion of comprehensive and accurate list of non-contraceptive benefits by FP method	.7
Side effect management	Inclusion of accurate management options for common side effects by FP method	.3

### Content review and analysis

Our medical advisor conducted a primary content review of each of the 11 tools, scoring each tool by content area, noting any omissions, inaccuracies, or incomplete descriptions, by FP method where applicable. Some content areas could be scored as “not applicable.” For example, a tool providing general information about FP methods does not verify reproductive intentions by design.

Two analysts with backgrounds in public health and expertise in FP and digital tools in low-and middle-income countries extracted information from the rubric applied by the medical advisor, quantifying the omissions, inaccuracies, and incomplete descriptions by family planning method for each key content area, and summarized the information in a master table (Table 3). Key content areas were assigned a categorical score of “green” (numerical value of + 1), “yellow” (numerical value of 0), or “red” (numerical value of -1) based on the following classification:

- Green (+ 1): Contains ≤ 1 omission, inaccuracy, or incomplete description
- Yellow (0): Contains 2–3 omissions, inaccuracies, or incomplete descriptions
- Red (-1): Contains ≥ 4 omissions, inaccuracies, or incomplete descriptions

**Table 3:** Omissions and errors in 11 tools by key content area

Content area	Reproductive intentions	List of all modern methods	Mechanism of action	Method effectiveness	Duration of protection	Return to fertility	Instructions for use	Dual protection	Discreetness	Side effects	Side effect management	Non-contraceptive benefits
# of tools with 0–1 omissions/errors	1	9	1	1	9	5	1	8	3	1	0	0
# of tools with 2–3 omissions/ errors	2	2	4	2	2	4	3	0	0	3	0	3
# of tools with ≥4 omissions/ errors	0	0	1	8	0	2	7	1	8	7	0	3
No content	8	0	5	0	0	1	0	2	3	0	11	5

This process was performed by the two reviewers for each tool. The reviewers discussed any inconsistencies in order to achieve consensus. One reviewer then performed a quality control audit by returning to the tool content to verify that the score matched the content. Using our final classifications, we then calculated numerical scores to answer two questions:

- How accurate and comprehensive was the content of each of the tools?
- How accurate and comprehensive was the content in each content area across all tools?

To calculate tool scores, we multiplied each key content area weight (Table 1) by the numerical assessment value, calculated the sum of these products, and divided this value by the total number of key content areas (i.e., 12) (Eq. 1).

1 $$\mathrm\Sigma\;(\mathrm{key}\;\mathrm{content}\;\mathrm{area}\;\mathrm{weight}\ast\mathrm{assessment}\;\mathrm{value})\;/\;(\#\;\mathrm{content}\;\mathrm{areas})$$

To score key content areas, we calculated the sum of numerical assessment values and divided this value by the total number of tools that received a score for that content area (i.e., 12) (Eq. 2).

2 $$\mathrm\Sigma\;(\mathrm{assessment}\;\mathrm{value})\;/\;(\#\;\mathrm{tools})$$

These numerical scores were then used to identify the most accurate and comprehensive content areas across the tools and to identify the strongest overall user-facing digital tools. Based on expert opinion and the pattern we saw across tools, we set a quality threshold: to be recommended for adaptation and scale up, tools could not have four or more errors in more than six content areas. In practice, this meant that tools scoring below -0.1 were deemed of insufficient quality and not recommended for adaptation and scale up.

One analyst reviewed the summary of omissions and inaccuracies included in the master table to populate insights shared in Table 5 relating to common omissions or inaccuracies. To provide examples of specific content, the analyst referred back to each tool.

## Results

**Fig. 1:**
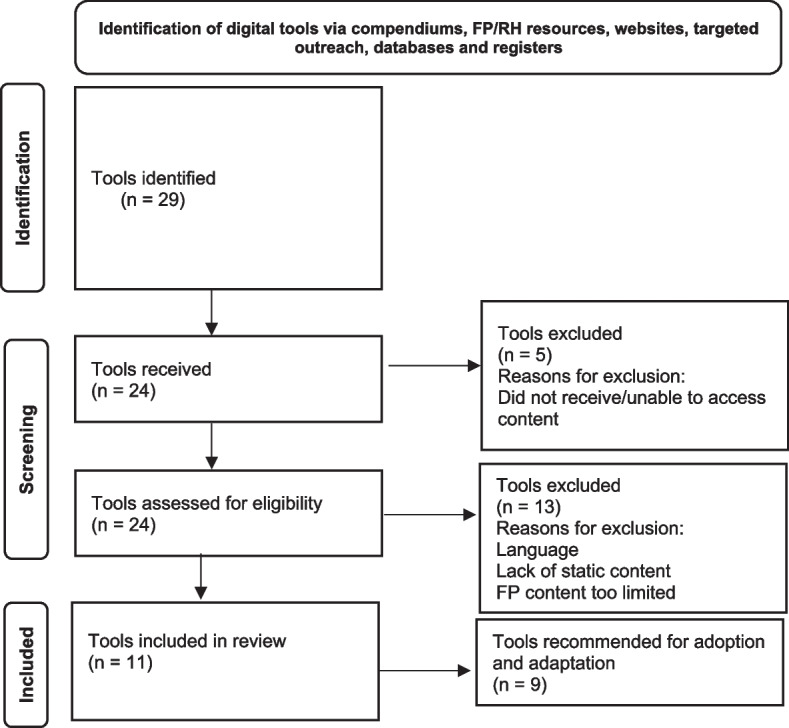
Landscaping user-facing tools

We contacted 29 tool owners about participating in the review and received content for 24 (83%) of the user-facing tools (see Fig. 1). The aforementioned team of two analysts reviewed each tool and applied the above-listed eligibility criteria, determining 11 tools were eligible for inclusion.

Of the tools included in the review, 8 were designed with youth in mind, while 3 were oriented to people of reproductive age. Table 3 synthesizes the findings from the analysis of key content areas across the tools.

Of the 12 key content areas we identified, five were included in each of the 11 tools; the tools addressed the remaining six content areas inconsistently or, in the case of side effect management, not at all (see Table 3). Four content areas were the most accurate and comprehensive: complete list of modern methods, duration of protection, dual method use, and return to fertility. The lowest scoring content areas were side effect management (not addressed in any of the tools), non-contraceptive benefits, effectiveness, side effects, and instructions for use.

The FP content of digital tools varied greatly in accuracy and comprehensiveness, with overall tool scores ranging from 0.28 to -0.52. Possible tool scores ranged from 0.82 to -82. Table 4 synthesizes the results from the analysis of the quality of each of the tools’ family planning content by tool.

**Table 4:** Scoring of FP content by tool

Tool	Reproductive intentions (.6)	List of all modern methods (1)	Mechanism of action (.6)	Method effectiveness (1)	Duration of protection (1)	Return to fertility (1)	Instructions for use (1)	Dual protection (1)	Discreetness (.7)	Side effects (1)	Side effect management (.3)	Non-contraceptive benefits (.7)	Tool score
10	NA	1	-0.6	1	1	0	0	1	0.7	0	-0.3	-0.7	0.28
6	.6	1	0	0	1	1	-1	1	-0.7	1	-0.3	-0.7	0.24
11	NA	1	-0.6	0	1	0	0	1	0.7	-1	-0.3	0	0.16
4	NA	1	-0.6	-1	1	1	1	-1	0.7	0	-0.3	-0.7	0.10
1	0	1	0	-1	1	0	-1	1	-0.7	-1	-0.3	0	-0.08
7	NA	0	0	-1	1	1	-1	1	-0.7	-1	-0.3	0	-0.09
2	0	1	-0.6	-1	1	1	-1	1	-0.7	-1	-0.3	-0.7	-0.11
3	NA	0	-0.6	-1	1	1	0	1	-0.7	-1	-0.3	-0.7	-0.12
5	NA	1	0.6	-1	0	-1	-1	1	-0.7	0	-0.3	-0.7	-0.19
9	NA	1	-0.6	-1	1	0	-1	-1	-0.7	-1	-0.3	-0.7	-0.39
8	NA	1	0	-1	0	-1	-1	-1	-.7	-1	-.3	-.7	-.52

We developed a table that included details of specific omissions and inaccuracies by content area for each tool. This information is synthesized in Table 5, which outlines trends in the inaccuracies and omissions across content areas in the tools we reviewed.

**Table 5:** Qualitative content area trends

Content area	Trends observed
Reproductive intentions	Instead of assuming tool users want to prevent pregnancy, tools should ask and clarify for how long. This is essential if the tool will recommend a contraceptive method or group of methods
List of all modern methods	Some tools were inconsistent when discussing all available modern methods or introduced inaccuracies in how they refer to certain methods
Mechanism of action	When addressed, most tools had more than one inaccuracy/omission, particularly for emergency contraceptive pills
Method effectiveness	Many tools do not distinguish between correct and consistent use (or perfect use) and typical use
Duration of protection	High-scoring tools often distinguished between short- and long-acting methods Four tools cited incorrect information about the duration of protection provided by implants and intrauterine devices available in their geographic location. This included outdated or/ shorter protection timeframes than those specified by clinical guidelines, or timeframes that were misaligned with the methods described
Return to fertility	More than half of the tools included two or more omissions or inaccuracies when describing return to fertility following the use of hormonal methods
Instructions for use	Instructions for oral contraceptive pills and emergency contraceptive pills frequently contained errors or omissions. Some tools failed to distinguish between combined oral contraceptives (COCs) and progestin only pills (POPs). Some instructions for IUD use omitted the need for a pelvic exam
Dual protection	Omission of guidance related to dual method use was most frequent in descriptions of fertility-awareness methods
Discreetness	There were missed opportunities to identify methods that can be used discreetly
Side effects	Information about the side effects of oral contraceptive pills was inaccurate or incomplete in ten of eleven tools. Some tools listed rare complications of IUD as side effects (e.g. uterine perforation)
Non-contraceptive benefits	Injectables, IUDs, and OCPs were the methods with the highest frequency of omissions or inaccuracies

## Discussion

Quality assurance is a foundational element of quality care for FP [6]. As FP self-care strategies, including digital, become more common, the FP community must determine how to translate principles of quality of care into these new contexts. Both the Digital Self-care Framework and Quality of Care Framework for Clients and Providers in the Delivery of Self Care lay out standards for client safety, indicating that client communication tools should be medically accurate and aligned with national and international guidelines [7, 8]. While this work focuses on accuracy and comprehensiveness, additional factors in the development of tool content are critical, including tailored design and content for the user’s context, literacy level, and preferences [9]. Because quality of care is an important factor that influences uptake and continued use of family planning methods, [10] the potential implications of inadequate or incorrect information in digital tools are far-reaching.

We assessed 11 digital, user-facing tools for quality of their FP content (by tool and by content area). Significant variation in quality was observed by content area, with most accurate and comprehensive areas being naming all available modern FP methods and duration of protection. The content areas with the lowest scores included side effect management and non-contraceptive benefits. Content areas that required more technical or clinical information, such as side effects, effectiveness, and instructions for use, were more likely to include errors in the tools we assessed. These findings resonate with other assessments that have noted that the FP/RH content of digital applications was not sufficiently complete or accurate [11, 12].

Misinformation or incomplete information about FP can contribute to dire consequences for those who are exposed to it, including unintended pregnancy and sexually transmitted infection. Accurate, comprehensive information is an essential component of informed decision-making about RH [13]. For example, when we consider effectiveness, it is important for potential users to know that effectiveness of some methods depends heavily on their ability to use those methods consistently and correctly. Therefore, tool content that presents perfect use statistics for effectiveness, without also including common use statistics, can be misleading. The tiered presentation of methods as most effective, moderately effective, and least effective can be considered as directive, subjective or inaccurate when presented out of the context of all methods [14].

The absence of some information in these digital tools represents a missed opportunity to educate individuals on important considerations and to address prevalent concerns. For example, three tools lacked comprehensive and accurate guidance related to dual method use. In the case of fertility awareness methods, users should be aware that they may still be at risk of STI/HIV (considering these methods include defined periods of unprotected sex when pregnancy is unlikely). As another example, no tools provided accurate information about non-contraceptive benefits such as improved menstrual regularity, reduced cramping and pain, and protection from certain types of gynecological cancers [15]. Provision of this knowledge is highly pertinent, as many users are drawn to non-contraceptive benefits when choosing a method and widespread misconceptions persist surrounding hormonal methods and cancer risk [15–17]. Non-contraceptive benefits should be covered systematically to reduce harmful myths and misconceptions and to help those who are deciding on an FP method to consider all advantages.

Side effects, both perceived and experienced, can lead to discontinuation or can prevent users from adopting a method [18–20]. Digital tools offer an opportunity to support informed choice and contraceptive continuation by providing users with accurate and comprehensive information on side effects before and after they adopt a method, so users know what to expect and are prepared if side effects occur. While discussion of rare complications can be an important component of comprehensive digital FP (and perhaps its own content area), presenting these as side effects is incorrect and can unnecessarily scare users. Digital tools are optimally designed to support self-care, particularly when they include after-care instructions and reassurance regarding common side effects (many of which can be managed by simple, over-the-counter medications or through other self-managed approaches).

Despite the emphasis on quality in existing frameworks for digital self-care, to our knowledge, no resources are available to assess whether the FP content of a given digital tool is of high quality. Global guidance documents [21–23] provide up-to-date family planning information with a high level of detail but they are not designed to serve as quality assurance tools, or to be easily converted into a user-friendly, digital format. Additionally, FP information is dynamic: new contraceptives are being added to the method mix and new evidence leads to changes in recommendations (e.g., the duration of protection of a method is extended, or drug interaction guidance is updated, or timing of initiation is changed). It can therefore be challenging to source and maintain accurate, sufficient, up-to-date content that is also relevant to the intended audience.

Our exercise resulted in a list of existing (as of April 2022) user-facing digital tools (see Appendix 1) which have high-quality FP content and can therefore be promoted or used by governments, donors and FP implementers. This exercise also led us to develop a simplified checklist from our original rubric (see Appendix 2) that can help these same stakeholders to assess and improve the FP content of user-facing digital tools that were not assessed as part of this exercise. The checklist can also be used as an outline for those developing new digital tools to ensure that key content areas are included; then, global FP resource documents can serve as a source for actual/updated technical content. While none of the tools we reviewed cited their content sources, we recommend doing so, as providing citations can assuage both users and clinicians, thereby making the tool more likely to be used as intended.

### Limitations

While we conducted an in-depth search of multiple databases to identify eligible tools to include in our review, we may have missed some existing tools. Also, our criteria limited the tools that were eligible for inclusion. For example, we did not consider tools that are available in languages other than English or French. However, the tools we reviewed are deployed in multiple contexts and 9 languages in total, which extends possible benefits of this analysis to populations speaking those languages. In addition, more complex tools, such as those with dynamic, user-generated, or influencer-generated content were not eligible for inclusion, yet these are an important source of FP information for some users and their content should also be reviewed and assessed. Tool owners had to agree to participate in the assessment and share their content with the study team. The assessment is based on the content that was present at time of review. Tool content may have changed since review via content updates. While a trained medical advisor assigned weights to content areas based on experience and evidence regarding the essential components of informed choice counseling and their relative importance from a biomedical perspective, this could be considered subjective and is likely different from individual user priorities.

## Conclusion

There has been rapid growth over the past decade in the number of digital tools that support FP knowledge and access. There has been similarly rapid growth in the enthusiasm for these types of tools—including as part of guidelines and efforts to support self-care—which has led to resources that can help interested governments, donors, and program implementers to understand the current evidence base and to identify existing digital FP tools. However, to our knowledge, there has not been similar attention paid to assessing the quality of the FP content that these digital tools provide. Supervision and quality assurance are routine parts of assessing and strengthening in-person FP. Now, the global FP community needs to incorporate ways to do the same for digital FP tools.

### Supplementary Information

Supplementary Material 1: Appendix 1_Brief_Results from FP content analysis.

Supplementary Material 2: Appendix 2_Quality Assessment Checklist_FP content.

## Data Availability

No datasets were generated or analysed during the current study.

## Abbreviations

FP: Family planning
FP/RH: Family planning and reproductive health
UNFPA: United Nations Population Fund
COCs: Combined oral contraceptives
POPs: Progestin-only pills
IUD: Intrauterine device
STI/HIV: Sexually transmitted infection/Human Immunodeficiency Virus
