# Supplementary material for: What’s inside matters: an assessment of the family planning content of digital self-care platforms
Source: Reprod Health. 2024 Jul 30;21:112. doi: 10.1186/s12978-024-01848-4 (PMC11290190; doi:10.1186/s12978-024-01848-4)
Supplement: Supplementary file 1 — Supplementary Material 1: Appendix 1_Brief_Results from FP content analysis. [file 12978_2024_1848_MOESM1_ESM.pdf]

So Many Tools; How to Choose?

Results from a Content Analysis of Family Planning Digital Platforms

USAID FROM THE AMERICAN PEOPLE

fhi360 THE SCIENCE OF IMPROVING LIVES

EVIHDAF

MAKERERE UNIVERSITY

psi Healthy lives. Measurable results.

Save the Children

So Many Tools; How to Choose?

Results from a Content Analysis of Family Planning Digital Platforms

BACKGROUND

The number of digital health interventions that provide access to family planning (FP) information and care in low- and middle-income countries has grown tremendously over the past decade. There are resources on **what interventions exist**—such as the **Digital Health Atlas** and the **Digital Health Compendium**—and **which interventions have evidence of impact**—such as the three digitally focused **High Impact Practice Briefs**. However, **no resources** currently exist that indicate **whether the content of the digital health interventions is of high quality.**

After conducting a broad landscape analysis to identify client-facing digital platforms designed to share information on FP, FHI 360 through the Research for Scalable Solutions project, assessed the quality—which we defined as **comprehensiveness and accuracy**—of the FP content of 11 tools that were currently in use, were available in English and French, and had static content we could access, which included SMS, chatbots, and websites. Technical staff at FHI 360 developed a rubric that outlined 11 content areas representing core elements of high-quality counseling (Box 1). We reviewed and scored each tool, assessing comprehensiveness and accuracy by content area. The static content we reviewed was sometimes only one component of a broader suite of digital tools offered by each program or organization. This brief is designed for anyone who may be interested in developing, implementing, or funding client-facing digital family planning interventions. It provides information as to where tool content was strongest and weakest, highlights key findings related to the content areas, and offers a list of tools recommended for adoption or adaptation. More details are available in an accompanying slide deck.

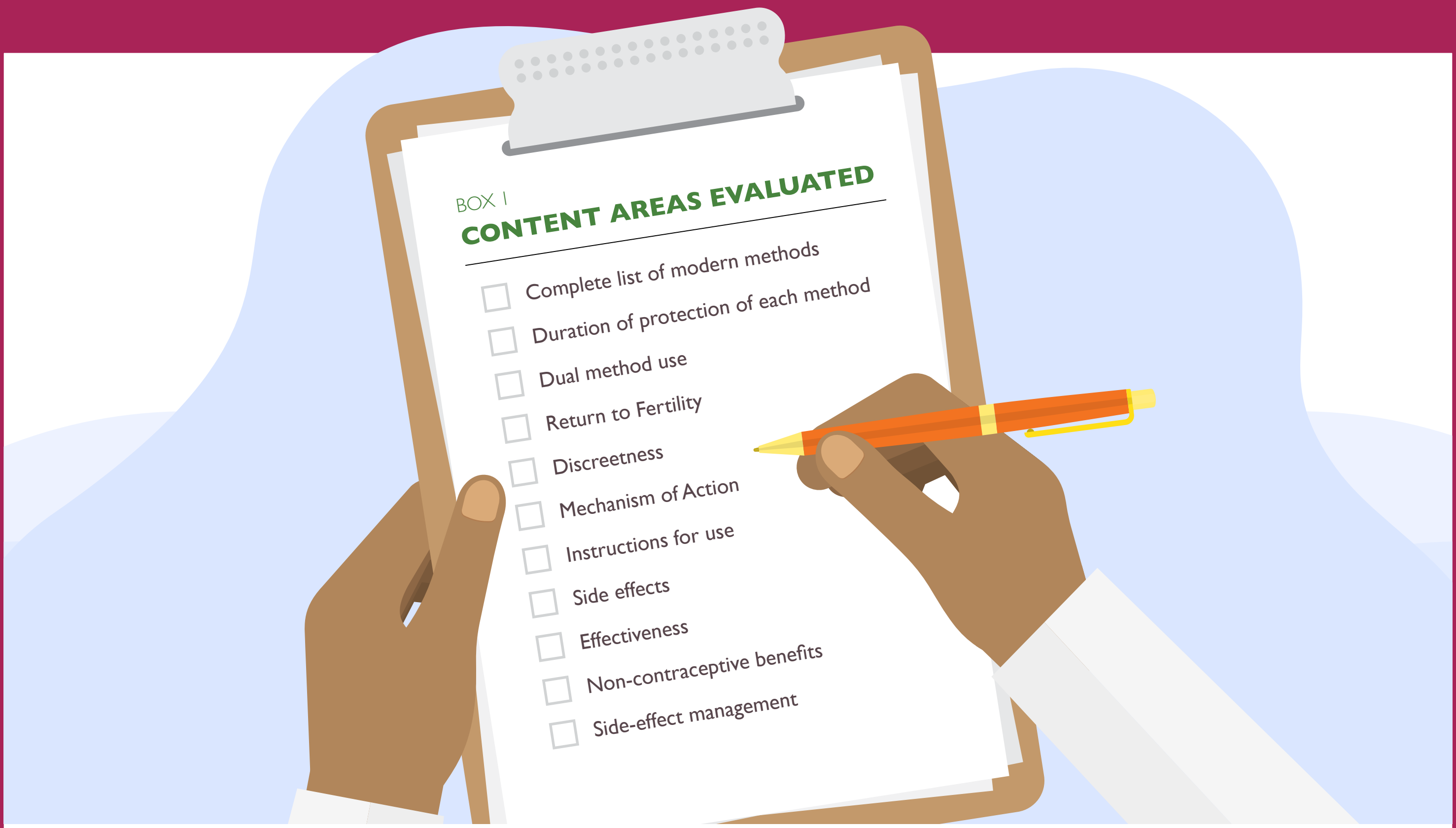

HERE'S WHAT WE FOUND

The following content areas are ordered from most to least accurate and comprehensive:

**Complete list of modern methods**	**All tools provided users with a complete list of methods available in their context. Only two tools included more than one omission or inaccuracy.** Some tools were inconsistent in the level of detail of the information provided for each method, which had the potential to bias users toward the methods with more comprehensive information.
**Duration of protection of each method**	**Information about the duration of protection tended to be accurate and complete and tools often distinguished between short- and long-acting and permanent methods.** Four tools included outdated information about the duration of protection of implants and intrauterine devices (IUDs), reinforcing the need to update content as global guidance changes.
**Dual method use**	**The tools we reviewed consistently noted that only condoms offer protection against HIV and sexually transmitted infections, and they encouraged their use in combination with other FP methods.** Omission of guidance to use dual protection was most frequent in descriptions of fertility-awareness methods.
**Return to Fertility**	**Five out of eleven tools provided comprehensive/accurate information about return to fertility.** Two tools included three or more omissions or inaccuracies when describing return to fertility following the use of hormonal methods.
**Discreetness**	**Only three tools contained accurate and comprehensive information related to whether certain methods may be used discreetly** (e.g., without a partner or parent's knowledge).

**Mechanism of action**	**Only one tool provided a complete and correct description of the mechanism of action of FP methods.** Explaining a method's mechanism of action can be important to combat misconceptions and misinformation that some FP methods are abortifacient.
**Instructions for use**	**Seven out of eleven tools included more than three inaccuracies in instructions for method use.** Instructions for oral contraceptive pills and emergency contraceptive pills frequently contained errors or omissions. For example, several tools emphasized that users should take the pill at the same time every day for it to be effective, though this is not the case for combined oral contraceptives, and progestin-only pills have a three-hour window. For adolescent users, a perceived need to adhere to a strict schedule may deter use. In addition, some instructions for IUD use omitted the need for a pelvic exam. This information often dissuades youth from adopting this method and, therefore, should be made very clear to potential users.
**Side effects**	**Only one of eleven tools included correct and complete information about side effects. Information about the side effects of oral contraceptive pills was inaccurate or incomplete in ten out of eleven tools.** For example, in addition to making periods more regular and predictable, it is valuable to add that periods also become lighter and shorter with this method. This feature is very important for young girls, especially those who have heavy menses the first few years after menarche. Given the risk of discontinuation due to side effects, the principle of informed choice, and potential application of digital tools for self-care, this is an important gap that should be addressed in future iterations of the tools we reviewed.
**Effectiveness**	**We limited our definition to inclusion of accurate typical use statistics, given that perfect use is relatively rare for user-dependent methods.** Eight out of eleven tools included more than three inaccuracies or omissions related to method effectiveness. Some tools described methods using tiers of effectiveness such as highly effective, effective, or not very effective, which could be subjective or were incorrect.
**Non-contraceptive benefits**	**The real or perceived benefits of using certain contraceptive methods** such as reduced risk of ovarian cancer or lighter periods—**were infrequently included and seven out of eleven tools had three or more omissions,** yet these benefits are often appealing both to youth and adult users of FP.
**Side-effect management**	**No tool shared information about side-effect management** (such as taking pain medication to relieve cramping), yet we know that difficulties in managing side effects can lead to discontinuation. Digital tools may be appropriate platforms to offer users information about managing side effects in real-time.

RECOMMENDED TOOLS

Our review assessed the inclusion of eleven key content areas of family planning information that are crucial to informed choice. We identified nine tools that contained sufficiently comprehensive and accurate information about FP. In selecting digital tools for implementation in a particular context, certain content areas may have elevated importance - for example, discreetness is of particular concern to unmarried adolescents in many regions. Each of the below-listed tools links clients to services or a method directly via a clinic/pharmacy-locator, a referral to NGO clinics, or e-pharmacy purchasing. **In line with the Principles for Digital Development to reuse and improve, these tools are well-suited for adoption or adaptation.**

✻ = evidence

9ja Girls BIG SISTA, developed by Population Services International (PSI) and implemented by Society for Family Health (SFH) under **A360**, interacts with Nigerian girls via a chatbot available on Facebook. Big Sista delivers content about family planning and reproductive health in bite size messages, including advantages, disadvantages, and frequently asked questions for each method. For a demo of the chatbot, send a message to **helloA360@psi.org**.

9JA GIRLS LIFE, LOVE, AND HEALTH GUIDEBOOK, developed by PSI and implemented by SFH under **A360's** 9ja girls, teaches Nigerian girls vocational skills and discusses a variety of topics related to love, relationships, and health. Classes are facilitated by young providers with a special commitment to girl-centered, non-judgmental healthcare. For more information, send a message to **helloA360@psi.org**.

ASKNIVI, by Nivi, Inc., is an interactive chat service that provides individuals in India, Kenya, Nigeria, and South Africa with information, recommended methods, and links to health services and products via WhatsApp and Facebook messenger. askNivi re-engages users after initial interaction to support contraceptive continuation. Nivi is sustained through partnerships. For more information, contact **Ben Bellows**. ✻

CHOICE, by MSI Reproductive Choices, is a web-based platform available to anyone, anywhere. "Choice" is a personalized contraceptive counselor that ranks FP methods based on user preferences, past experience, and medical eligibility, allowing users to compare options while considering the advantages and disadvantages of each method. The straightforward content on the website can be easily leveraged and applied in diverse programming contexts—and MSI Reproductive Choices welcomes others to use it. For more information or to access the tool's content for adaptation, contact **Rachel Misra** .

COUNSELING FOR CHOICE CHATBOT, developed by PSI, is a chatbot designed to provide FP and reproductive health information and access to young people and adults. In Cote d'Ivoire, the first implementation country, the chatbot – locally called "Tante Gabi" – asks a series of questions to understand user preferences and needs, then provides a list of three suggested FP methods that best fit those stated needs; Tante Gabi then links users to local clinics and provides a referral code. PSI is using the tool content as the basis for expansion in West Africa in the near term and plans to continue growing its footprint into the future. More information is available **here**.

CYBERRWANDA, developed by YLabs, was co-designed with Rwandan youth to provide information and direct-to-consumer services via edutainment (comics), a website, a native app, and linkage to youth-friendly services. CyberRwanda works within and outside of schools to provide information on topics ranging from puberty to FP, relationships, and goal setting. For more information or to discuss adaptation, please contact **Rebecca Hope** and **Emmyson Gatare**.

LOVEMATTERS, by RNW Media, provides pleasure-positive, locally adapted information about reproductive health, menstrual health and hygiene, and FP as well as links to services for youth across seven platforms in a variety of languages via websites and accompanying social media pages. LoveMatters is designed for replication at scale and welcomes partnerships. For more information, please contact **info@rnw.org**.

M4RH, by FHI 360, delivers simple, streamlined content about FP methods to women, men, and youth, as well as a clinic locator option. It has been implemented primarily as an SMS service, but the messages have also been adapted to unstructured supplementary service data (USSD) and interactive voice response (IVR). Anyone interested in adopting or adapting the content can access it via a simple license agreement, which more than 10 organizations have already done. It has also been adopted into national programs in both Tanzania and Rwanda.

TUNEME, created by Praekelt Foundation with funding from the United Nations Population Fund (UNFPA) and the Ford Foundation, delivers locally relevant content on FP, menstrual health and hygiene, puberty, relationships, COVID-19, and more via websites for youth in Botswana, Lesotho, Malawi, Namibia, Eswatini, Zimbabwe, and Zambia. Built with scale in mind, please contact **Renata Tallarico** to use TuneMe in your context.

This document is made possible by the support of the American people through USAID. Research for Scalable Solutions (R4S) is a global project funded by USAID and led by FHI 360 in partnership with Evidence for Sustainable Human Development Systems in Africa (EVIHDAF), Makerere University School of Public Health in Uganda (MakSPH), PSI, and Save the Children (STC). The contents of this brief are the sole responsibility of FHI 360 and do not necessarily reflect the views of USAID or the United States Government.

Recommended citation: Brittingham Sarah, Zan Trinity, Yacobson Irina. So many tools; how to choose? Results from a content analysis of family planning digital platforms (brief). Durham (NC): **Research for Scalable Solutions**/FHI 360;2021.
