# Supplementary material for: What’s inside matters: an assessment of the family planning content of digital self-care platforms
Source: Reprod Health. 2024 Jul 30;21:112. doi: 10.1186/s12978-024-01848-4 (PMC11290190; doi:10.1186/s12978-024-01848-4)
Supplement: Supplementary file 2 — Supplementary Material 2: Appendix 2_Quality Assessment Checklist_FP content. [file 12978_2024_1848_MOESM2_ESM.pdf]

Quality Assessment Checklist for Client-facing Family Planning Content in Digital Tools

This is designed to be a user-friendly checklist that will guide you through the steps to assess and improve the family planning (FP) content of your digital tool. The need for an assessment checklist was identified in 2021 during a **review** of the FP content of 11 digital tools that were currently in use, conducted by FHI 360 through the Research for Scalable Solutions project. The review assessed the comprehensiveness and accuracy of 13 FP content areas and identified opportunities where tool developers could improve. A full report, including FP content excerpts is available **here**.

This checklist walks users through the process to assess and improve their tool by content area. For each content area, there is space to verify the presence of information (by contraceptive method, where applicable). We've developed tips to address omissions and inaccuracies frequently observed during the review and select key messages to address those omissions and inaccuracies.

The checklist identifies 13 FP content areas necessary for high quality, comprehensive content that supports informed decision making.

This tool does not provide the key messages or technical content by family planning methods and/or content areas, nor does it address method eligibility criteria rather, use **Family Planning: A Global Handbook for Providers** or **Medical Eligibility Criteria for Contraceptive Use** as the content resource for all sections. Additional resources (where applicable) will be mentioned by content area.

While this checklist tool focuses on the accuracy and comprehensiveness of the FP content, the information should be person-centered, easy to access/understand, and culturally relevant. Conduct usability tests of the interface design and examine whether users correctly interpret/use the information.

Given that young people are majority users of FP/RH digital tools globally, we've signaled recommendations that are especially relevant to youth audiences with this icon.

INSTRUCTIONS

To assess the family planning content of your digital tool, we recommend that you follow the below-listed steps. Users of this tool often worked in teams of 2 and took from 30 minutes to 8 hours.

STEP 1: To facilitate your review, extract the family planning content of your tool and transfer it to an easily editable software (e.g., MS Word). Turn on the track changes feature to visualize the edits you make and communicate them to your team members. This will make it easier to implement the changes later.

STEP 2: Open up the technical resource that will inform your content assessment. We recommend **Family Planning: A Global Handbook for Providers**, which contains information for each content area assessed in this checklist.

STEP 3: Tick which of these 13 content areas are included in the tool:

- ❑ Reproductive intentions
- ❑ Complete list of modern methods
- ❑ Mechanism of action
- ❑ Duration of protection
- ❑ Effectiveness
- ❑ Return to fertility
- ❑ Discreetness
- ❑ Dual protection/dual method use
- ❑ Side effects
- ❑ Non-contraceptive benefits
- ❑ Instructions for use
- ❑ Side-effect management
- ❑ Where to obtain the method

Note: If the tool addresses a content area for any family planning method, it should address that content area for all the FP methods.

STEP 4: Use the **table**, Content Areas by Weight, at the end of this document to record and justify the omission of any content area listed above. Strike through the relevant content area throughout the body of this document.

STEP 5: Verify which family planning methods are available in the context in which the tool is deployed. Refer to local family planning guidelines and service delivery guidelines (if available and updated within the last five years) disseminated by the MoH — for a list of methods as well as the types of facilities where the various methods are available. In the list below, tick the methods that are available to the tool users; skip or strikethrough unavailable methods in the subsequent sections of the checklist.

- ❑ Combined oral contraceptive pill (COCs)
- ❑ Progestin-only pills (POPs)
- ❑ Emergency contraceptive pills (ECPs)
- ❑ Injectable(s) (note if more than one type of Injectable(s) is available and include accordingly)
- ❑ Combined patch
- ❑ Combined vaginal ring
- ❑ Implant(s) (note if both 1-rod and 2-rod Implant(s) are available and include accordingly)
- ❑ Copper-bearing intrauterine device (Cu-IUD, IUCD)
- ❑ Levonorgestrel intrauterine device (hormonal IUD)
- ❑ Tubal ligation/Female sterilization
- ❑ Vasectomy/Male sterilization
- ❑ Male condoms
- ❑ Female condoms
- ❑ Fertility awareness methods (FAM)/Standard Days Method (SDM)
- ❑ Lactational Amenorrhea Method (LAM)

Optional methods for inclusion (Note: As commonly used, these have very high failure rates; additionally, spermicides increase the risk of acquiring HIV if exposed. However, for some clients, these may be the only options available/acceptable):

- ❑ Spermicides
- ❑ Diaphragms
- ❑ Withdrawal

Quality Assessment Checklist

Name of tool:

Date reviewed:

Staff member(s) who completed review:

Sections/pages of tool reviewed:

List of locations where tool is implemented:

Target audience for tool:

Objective of tool / desired outcome in target population:

TIP

Many tools assume that adolescents want to prevent pregnancy, but it is best to ask.

1. Reproductive intentions

The length of desired pregnancy prevention is a factor in decision-making but should not limit contraceptive choice. If desired, short-acting methods can be used to prevent pregnancy for many years and long-acting methods can be used to prevent pregnancy for a year or two. All reversible methods for which the user who wants to delay pregnancy is medically eligible should be offered.

Verify that if the tool will recommend a contraceptive method or a group of methods, it clarifies the user's reproductive intentions — the desire/plan to have (or not) a child or children, as well as the timing.

2. Complete list of modern methods

Verify that all modern methods that are available to the target population in the context in which the tool is deployed are named (see step 5 on preceding page).

- ❑ Combined oral contraceptive pill (COCs)
- ❑ Progestin-only pills (POPs)
- ❑ Emergency contraceptive pills (ECPs)
- ❑ Injectable(s)
- ❑ Combined patch
- ❑ Combined vaginal ring
- ❑ Implant(s)

TIPS

Differentiate between combined oral contraceptives (COCs) and progestin-only pills (POPs), as the instructions for use are quite different (for more on this, consult section 7 below). This is especially relevant for adolescent-facing tools.

If a method has multiple names (e.g. POPs are often called mini pills), share both.

- ❑ Copper-Bearing Intrauterine Device (Cu-IUD, IUCD)
- ❑ Levonorgestrel Intrauterine Device (hormonal IUD)
- ❑ Tubal ligation/Female sterilization
- ❑ Vasectomy/Male sterilization
- ❑ Male condoms
- ❑ Female condoms
- ❑ Fertility awareness methods/Standard Days Method
- ❑ Lactational Amenorrhea Method (LAM)
- ❑ Spermicides
- ❑ Diaphragms
- ❑ Withdrawal

TIP

Use simple language. For example, instead of "prevents ovulation," use "prevents the release of an egg by the ovary during a woman's menstrual cycle," or instead of "prevents fertilization," use "prevents sperm from uniting with an egg."

3. Mechanism of action

Misconceptions related to the mechanism of action can steer users away from certain methods. This often occurs in religious contexts, where users may have the misconception that an FP method acts as an abortifacient.

In our review, the description of the mechanism of action for Emergency contraceptive pills (ECPs) was often inaccurate. POPs, IUDs, Combined vaginal ring, and patch also included inaccuracies; check these carefully against an up-to-date content source, such as the **Family Planning Handbook**

Verify that the mechanism of action is shared for each method.

- ❑ Combined oral contraceptive pill (COCs)
- ❑ Progestin-only pills (POPs)
- ❑ Emergency contraceptive pills (ECPs)
- ❑ Injectable(s)
- ❑ Combined patch
- ❑ Combined vaginal ring
- ❑ Implant(s)
- ❑ Copper-Bearing Intrauterine Device (Cu-IUD, IUCD)
- ❑ Levonorgestrel Intrauterine Device (hormonal IUD)
- ❑ Tubal ligation/Female sterilization
- ❑ Vasectomy/Male sterilization
- ❑ Male condoms
- ❑ Female condoms
- ❑ Fertility awareness methods/Standard Days Method

- ❑ Lactational Amenorrhea Method (LAM)
- ❑ Spermicides
- ❑ Diaphragms
- ❑ Withdrawal

TIP

Many tools elect to indicate duration of protection only for methods that are not required at each intercourse. Still, it is good to emphasize duration of protection for these methods (e.g., protect for one sexual act only), especially if the audience for the tool are adolescents/young people.

4. Duration of protection

Verify that your tool indicates the duration of protection of all methods:

- ❑ Combined oral contraceptive pill (COCs)
- ❑ Progestin-only pills (POPs)
- ❑ Emergency contraceptive pills (ECPs)
- ❑ Injectable(s)
- ❑ Combined patch
- ❑ Combined vaginal ring
- ❑ Implant(s)
  Note: In our review, some tools included outdated information about the duration of protection for implant(s). The current range is 3–5 years, depending on the type of implant.
- ❑ Copper-Bearing Intrauterine Device (Cu-IUD, IUCD)
  Note: In our review, IUDs contained the most frequent inaccuracies. The most common copper IUCD provides protection against pregnancy for up to 12 years (many tools listed 10 years based on the manufacturer labeling). This information can be shared as a range if multiple options are available or if the tool will be deployed across multiple contexts.
- ❑ Levonorgestrel Intrauterine Device (hormonal IUD)
- ❑ Tubal ligation/Female sterilization
- ❑ Vasectomy/Male sterilization
- ❑ Male Condoms
- ❑ Female Condoms
- ❑ Fertility awareness methods/Standard Days Method
- ❑ Lactational Amenorrhea Method (LAM)
- ❑ Spermicides
- ❑ Diaphragms
- ❑ Withdrawal

5. Method effectiveness

In our review, we frequently noted inaccuracies when tools described effectiveness or failure rates in tiers (e.g., less effective, more effective). Instead, consider using a visual representation, such as **this chart in the FP Handbook**.

Verify that the tool differentiates between method effectiveness as commonly used (i.e., typical use) and effectiveness when method is used consistently and correctly (i.e., perfect use). This is particularly important for all short-acting methods because the effectiveness of these methods depends greatly on ability of clients to use them correctly. Sometimes tools focus on typical use only, however, discussing perfect use and what it entails may help clients to maximize effectiveness. Opportunities to maximize effectiveness will be shared by method below, applicable.

- ❑ Either use a percentage (e.g. about 93% effective) OR the same denominator for each method (e.g., about 7 pregnancies per 100 women using the method over the first year) so that users can easily compre methods.

- ❑ **Combined oral contraceptive pill (COCs)**
  - ❑ Typical use
  - ❑ Perfect use

    Note: Remind users to take pills on schedule to maximize effectiveness.

- ❑ **Progestin-only pills (POPs)**
  - ❑ Typical use
  - ❑ Perfect use

    Note:
    - *Remind users to take pills on schedule to maximize effectiveness.*
    - *We recommend including information on effectiveness among both non-breastfeeding women and breastfeeding women as POPs are more effective in women who breastfeed.*

- ❑ **Emergency contraceptive pills (ECPs)**

  Note: Because ECPs are taken only after unprotected sex and are not intended for continuous use, their effectiveness can not be measured as "perfect" and "typical" like methods that are taken on a daily, monthly, or weekly schedule. As long as ECPs are taken within the first 120 hours after unprotected sex, they are used correctly. However, it is important to discuss with potential users that they can maximize effectiveness by taking ECPs as soon as possible after unprotected sex (e.g., taking ECPs within 24 hours can provide greater effectiveness than taking them later within the 120 hour timeframe).

- ❑ **Injectable(s)**
  - ❑ Typical use
  - ❑ Perfect use

  Note: Remind users to come for re-injection on time to maximize effectiveness.

- ❑ **Combined patch**
  - ❑ Typical use
  - ❑ Perfect use

  Note: Remind users to replace the patch on time to maximize effectiveness.

- ❑ **Combined vaginal ring**
  - ❑ Typical use
  - ❑ Perfect use

  Note: Remind users to replace the ring on time to maximize effectiveness.

- ❑ **Implant(s)**
  - ❑ Typical use
  - ❑ Perfect use

  Note: Remind users that effectiveness is the same in typical and perfect use and there is nothing the user needs to do or remember to maximize effectiveness.

- ❑ **Copper-Bearing Intrauterine Device (Cu-IUD, IUCD)**
  - ❑ Typical use
  - ❑ Perfect use

  Note: Remind users that effectiveness is practically the same in typical and perfect use and there is nothing the user needs to do or remember to maximize effectiveness.

- ❑ **Levonorgestrel Intrauterine Device (hormonal IUD)**
  - ❑ Typical use
  - ❑ Perfect use

  Note: Remind users that effectiveness is practically the same in typical and perfect use and there is nothing the user needs to do or remember to maximize effectiveness.

- ❑ **Tubal ligation/Female sterilization**
  - ❑ Typical use
  - ❑ Perfect use

  Note: Remind users that effectiveness is the same in typical and perfect use and there is nothing the user needs to do or remember to maximize effectiveness.

TIP

For methods where perfect and typical use are the same, such as the IUD, consider adding “without the user having to do or remember anything.”

- ❑ **Vasectomy/Male sterilization**
  - ❑ Typical use
  - ❑ Perfect use

    Note: Remind users that effectiveness is practically the same in typical and perfect use, but vasectomy is not fully effective for 3 months after the procedure. To maximize effectiveness, another method of contraception should be used for the first three months.

- ❑ **Male condoms**
  - ❑ Typical use
  - ❑ Perfect use

    Note: Emphasize that in order to maximize effectiveness, condom should be used correctly with every act of sexual intercourse.

- ❑ **Female condoms**
  - ❑ Typical use
  - ❑ Perfect use

    Note: Emphasize that in order to maximize effectiveness, condom should be used correctly with every act of sexual intercourse.

- ❑ **Fertility awareness methods/Standard Days Method (SDM)**
  - ❑ Typical use
  - ❑ Perfect use

    Note: Emphasize that being able to avoid unprotected sex during the days when pregnancy is likely to occur maximizes effectiveness.
    Note: In our review, some tools advised against fertility awareness methods due to high failure rates. However, fertility awareness methods, SDM in particular, have similar failure rates to the male condom, in both typical and perfect use.

- ❑ **Lactational Amenorrhea Method (LAM)**
  - ❑ Typical use
  - ❑ Perfect use

    Note: Emphasize that adhering to all three LAM criteria maximizes effectiveness.

- ❑ **Spermicides**
  - ❑ Typical use
  - ❑ Perfect use

    Note: Explain that spermicides are only moderately effective even when used perfectly and even less effective in common use. To achieve moderate effectiveness, they should be used consistently and correctly with every act of sexual intercourse.

- ❑ **Diaphragm**
  - ❑ Typical use
  - ❑ Perfect use

  Note: Explain that diaphragm is only moderately effective even when used perfectly and even less effective in common use. To achieve this moderate effectiveness, it should be used consistently and correctly with every act of sexual intercourse.

- ❑ **Withdrawal**
  - ❑ Typical use
  - ❑ Perfect use

  Note: Explain that to maximize effectiveness, man should be able to withdraw his penis from the vagina before he ejaculates. This may be hard to achieve and requires practice. This is why in typical use withdrawal is one of the least effective methods of contraception.

TIP

Avoid using terms such as "immediately reversible," which may not be clear; instead use: "after stopping the method, the ability to get pregnant returns without delay."

6. Return to fertility

In our review, we noted inaccuracies in information about return to fertility for hormonal methods. Given that many women have misperceptions and fears about the return to fertility after use of contraceptive methods, clearly state that among reversible methods, only progestin-only Injectable(s) may delay return to fertility. For all other methods, including hormonal contraceptives such as COCs, POPs, Combined patch, Combined vaginal ring, and hormonal IUD, clearly state there is no delay.

Ensure your tool includes accurate information about return to fertility for each method.

- ❑ Combined oral contraceptive pill (COCs)
- ❑ Progestin-only pills (POPs)
- ❑ Emergency contraceptive pills (ECPs)
- ❑ Injectable(s)

  Note: Users of Injectable(s) may experience a delay in return to fertility (for 4–9 months on average and up to 1 year).
- ❑ Combined patch
- ❑ Combined vaginal ring
- ❑ Implant(s)
- ❑ Copper-Bearing Intrauterine Device (Cu-IUD, IUCD)
- ❑ Levonorgestrel Intrauterine Device (hormonal IUD)
- ❑ Tubal ligation/Female sterilization
- ❑ Vasectomy/Male sterilization

❑ Male condoms

❑ Female condoms

❑ Fertility awareness methods/Standard Days Method

❑ Lactational Amenorrhea Method (LAM)
Note: Indicate that fertility is expected to return after LAM is stopped (or when any one of LAM criteria expires), but will depend on how much the woman continues to breastfeed.

❑ Spermicides

❑ Diaphragms

❑ Withdrawal

7. Discreetness

Many women are interested in using a method but may need to keep their use private from a partner or/and family members for a variety of reasons.

Ensure your tool describes the discreetness of each method. Below are observations about several methods which can be used discreetly but were not identified as discreet methods in the content review.

❑ **Combined oral contraceptive pill (COCs)**
Note: The pill is one of the least discreet methods, as someone can see when you take the pill and/or see where you store them, especially when youth live with their parents or partner.

❑ **Progestin-only pills (POPs)**
Note: The pill is one of the least discreet methods, as someone can see when you take the pill and/or see where you store them, especially when youth live with their parents or partner.

❑ **Emergency contraceptive pills (ECPs)**
Note: The pill is one of the least discreet methods, as someone can see when you take the pill and/or see where you store them, especially when youth live with their parents or partner.

❑ **Injectable(s)**
Note: Depending on the target audience for the tool, consider mentioning that partners may notice/question changes in bleeding pattern, e.g. "Injectable(s) are discreet, as no one can see that you are using this method; however, partners may sometimes notice/question changes in your bleeding pattern)."

❑ Combined patch

❑ Combined vaginal ring

TIPS

Instead of the word discreet, say "no one can see that you are using it," or "it will not be obvious to others that you are using x."

If not in a separate section, this information can be paired with information about advantages (and disadvantages for those that are indiscreet). Consider indicating which methods are woman-controlled and which require partner cooperation.

- ❑ Implant(s)
- ❑ Copper-Bearing Intrauterine Device (Cu-IUD, IUCD)
- ❑ Levonorgestrel Intrauterine Device (hormonal IUD)
- ❑ Tubal ligation/Female sterilization
- ❑ Vasectomy/Male sterilization
- ❑ Male Condoms
  Note: Cannot be used without partner's cooperation
- ❑ Female Condoms
  Note: Cannot be used without partner's cooperation
- ❑ Fertility awareness methods/Standard Days Method
  Note: Cannot be used without partner's cooperation
- ❑ Lactational Amenorrhea Method (LAM)
  Note: Depending on the target audience, consider noting that this method "can be used for pregnancy prevention without others knowing."
- ❑ Spermicides
- ❑ Diaphragms
- ❑ Withdrawal
  Note: Cannot be used without partner's cooperation

8. Dual protection and dual method use

The term 'dual protection' describes methods' ability to protect against both unwanted pregnancy and HIV and other sexually transmitted infections (STIs). Verify that each method includes an accurate dual method use message, clarifying if the method does/does not provide protection against HIV or STIs. Note that only male and female condoms supply dual protection.

Example: Only correct and consistent use of condoms can prevent the spread of HIV and STIs. Using a condom in addition to an effective non-barrier contraceptive method provides better protection from both pregnancy and STI/HIV.

- ❑ Combined oral contraceptive pill (COCs)
- ❑ Progestin-only pills (POPs)
- ❑ Emergency contraceptive pills (ECPs)
- ❑ Injectable(s)
- ❑ Combined patch
- ❑ Combined vaginal ring
- ❑ Implant(s)
- ❑ Copper-Bearing Intrauterine Device (Cu-IUD, IUCD)

- ❑ Levonorgestrel Intrauterine Device (hormonal IUD)
- ❑ Tubal ligation/Female sterilization
- ❑ Vasectomy/Male sterilization
- ❑ Fertility awareness methods/Standard Days Method
- ❑ Lactational Amenorrhea Method (LAM)
- ❑ Spermicides
- ❑ Diaphragms
- ❑ Withdrawal

9. Side effects

It is recommended to cover common side effects for each method. This will help potential users make informed choices. It will also reinforce information that was shared during a counseling session with a provider that a user may be less likely to recall.

The review of tools revealed that information about the side effects of methods, including bleeding and other menstrual changes, is often inaccurate or incomplete. The examples below provide correct messages for some of the more common errors observed in our review.

Verify that each method includes accurate and comprehensive information about side effects.

- ❑ Combined oral contraceptive pill (COCs)
  COCs make periods more regular, lighter and less painful, which could be very attractive to youth.
- ❑ Progestin-only pills (POPs)
- ❑ Emergency contraceptive pills (ECPs)
- ❑ Injectable(s)
- ❑ Combined patch
- ❑ Combined vaginal ring
- ❑ Implant(s)
  Note: Include common side effects such as bleeding changes as well as non-menstrual side effects such as nausea, abdominal pain, dizziness, and mood changes.
- ❑ Copper-Bearing Intrauterine Device (Cu-IUD, IUCD)
  Note: Side effects include 1) heavier and longer periods; 2) menstrual cramping.
  Note: Avoid classifying complications, such as uterine perforation, which is very rare, as a side effect; this may scare possible users unnecessarily.

TIPS

As user may seek reassurance in a digital tool to support contraceptive continuation, it can be helpful to clarify that common side effects are not harmful.

Do not include rare complications as side effects. This can unnecessarily scare users.

Be specific. Instead of, for example, "may experience changes to the menstrual cycle," state "Bleeding changes are a common side effect of injectable contraceptives. For the first several months of use, you may see your menses becoming irregular or longer than usual. With time, you may experience infrequent bleeding or no bleeding at all. This is normal and not harmful. Other common side effects may include headaches, dizziness, mood changes, and weight gain. Most women will experience some, but not all, of these side effects."

- ❑ Levonorgestrel Intrauterine Device (hormonal IUD)
  Note: Don't classify rare complications, such as pelvic perforation with the IUD, as a side effect; this may scare possible users unnecessarily.
- ❑ Tubal ligation/Female sterilization
- ❑ Vasectomy/Male sterilization
- ❑ Male condoms
  Note: Don't classify the possibility of a latex allergy and subsequent adverse reaction, which is extremely rare and considered a complication, as a side effect.
- ❑ Female condoms
- ❑ Fertility awareness methods/Standard Days Method
- ❑ Lactational Amenorrhea Method (LAM)
- ❑ Spermicides
- ❑ Diaphragms
- ❑ Withdrawal

TIP

Instead of stating "protects against some forms of cancer," share *which* cancers.

10. Non-contraceptive benefits

Many women are drawn to non-contraceptive benefits, for example improved menstrual regularity, reduced cramping and pain, or protection from certain types of cancers. There is also a widespread misconception that hormonal methods cause cancer. Yet this information was infrequently included in the tools we reviewed. Covering these benefits in detail can help to reduce harmful myths and misconceptions.

Verify that the tool shares complete and comprehensive information about non-contraceptive benefits. Included below are some of the most salient key messages related to non-contraceptive benefits. Consult the FP Handbook for comprehensive information.

- ❑ Combined oral contraceptive pill (COCs)
  Note: In addition to making periods more regular and predictable, periods also become lighter, shorter, and less painful with this method.
  This feature is very important for young girls, especially those who have heavy menses the first few years after menarche.
  Only COCs, not POPs, make the menstrual cycle more regular and lighter. Other important benefits include protection against cancer of the ovaries, cancer of the lining of the uterus, Ovarian cysts, iron-deficiency anemia and reduction of reduction of pelvic pain and bleeding in women with endometriosis and reduction of sickle cell crises in women with sickle cell anemia.

- ❑ Progestin-only pills (POPs)
- ❑ Emergency contraceptive pills (ECPs)
- ❑ Injectable(s)
  Note: Provide protection against cancer of the lining of the uterus (endometrial cancer), uterine fibroids, and iron-deficiency anemia.
  Note: Reduce pelvic pain and bleeding in women with endometriosis and sickle cell crises in women with sickle cell anemia.
- ❑ Combined patch
- ❑ Combined vaginal ring
- ❑ Implant(s)
- ❑ Copper-Bearing Intrauterine Device (Cu-IUD, IUCD)
  Note: Provides protection against endometrial and cervical cancers and reduces risk of ectopic pregnancy.
- ❑ Levonorgestrel Intrauterine Device (hormonal IUD)
  Note: Provides protection against endometrial and cervical cancers and iron-deficiency anemia. Reduces risk of ectopic pregnancy, as well as pain and bleeding in women with endometriosis and menstrual cramps.
- ❑ Tubal ligation/Female sterilization
- ❑ Vasectomy/Male sterilization
- ❑ Male condoms
  Note: Provide protection from conditions caused by STIs including cervical cancer, PID, and male/female infertility.
- ❑ Female condoms
  Note: Provide protection from conditions caused by STIs including cervical cancer, PID, and male/female infertility.
- ❑ Fertility awareness methods/Standard Days Method
- ❑ Lactational Amenorrhea Method (LAM)
- ❑ Spermicides
- ❑ Diaphragms
- ❑ Withdrawal

11. Instructions for use

In our review, oral contraceptive pills, IUDs, fertility awareness methods, and condoms were most likely to contain errors or omissions related to instructions for use, so check these carefully against an up-to-date content source.

Depending on the purpose of the tool, instructions for how to use the method may be very basic (e.g., indicating that COCs should be taken every day and relying on provider to give more detailed instructions on what to do if pills are missed, etc.) or instructions may be more detailed. Verify that the level of information provided is consistent across methods and reflects the purpose of the tool (basic education about contraceptive options/demand generation vs. more detailed information to facilitate informed choice).

Verify the presence of accurate instructions for use for each method. Key messages related to those issues/methods are included below:

- ❑ Combined oral contraceptive pill (COCs):
  Note: Distinguish between POPs and COCs, as only POPs must be taken approximately at the same time every day.
  Note: Include instructions for what to do if 1–2 pills are missed, or if 3+ pills are missed.
- ❑ Progestin-only pills (POPs)
  Note: Distinguish between POPs and COCs; POPs should be taken within a three-hour window every day. Include instructions on what to do if a pill is taken 3 or more hours late.
- ❑ Emergency contraceptive pills (ECPs)
  Note: Some tools mention ranges of time after sex that ECPs can be used (or rely on outdated guidance of 72 hours, or simply say to use emergency contraception as soon as possible after unprotected sex), but should instead state clearly: "use within 120 hours/5 days after unprotected sex, but the sooner it is taken the more effective it will be." In addition to describing that the next menses after taking EC pills could come a few days early or late, it is valuable to add that if menses are more than a week late, one should see a provider to rule out pregnancy.
- ❑ Injectable(s)
  Note: If more than one type of Injectable(s) is available, indicate correct use for each type.
- ❑ Combined patch
  Note: Indicate what to do if the patch is not replaced on time, becomes partially detached, or falls off.

TIPS

Over-simplification can lead to inaccuracies.

Use clear, descriptive language.

Instead of:

- "low frequency use," say: "doesn't require daily use."
- "the fertile window," say: "the days when you are most likely to get pregnant
- "peri-coital" say: "shortly before or after the time of sex."
- "When used properly," state "when used consistently and correctly" or "used correctly every time a couple has sex"

Recommended sources:

- **Selected Practice Recommendations for Contraceptive Use**
- **Family Planning: A Global Handbook for Providers**
- Manufacturer guidance

❑ Combined vaginal ring

Note: Indicate what to do if a new ring was not inserted on time or was taken out for 48 hours or more.

❑ Implant(s)

❑ Copper-Bearing Intrauterine Device (Cu-IUD, IUCD):

Note: An IUD insertion requires a pelvic exam, as this may be an important decision-making point for some potential users. Do not include outdated guidance encouraging users to check for IUD strings by inserting their finger into the vagina; this is no longer recommended as women can accidentally pull the strings, dislodging the IUD.

❑ Levonorgestrel Intrauterine Device (hormonal IUD):

Note: It is important to indicate that an IUD insertion requires a pelvic exam, as this may be an important decision-making point for some potential users. Many tools included outdated guidance that users should be checking if IUD is in place by inserting finger into the vagina to feel the strings; this is no longer recommended as women can accidentally pull the strings, dislodging the IUD.

❑ Tubal ligation/Female sterilization

❑ Vasectomy/Male sterilization

❑ Male condoms

Note: Do not include outdated guidance to pinch the tip of the condom. While there is no harm in squeezing the tip of the condom, the WHO no longer requires this step, as it was demonstrated that not squeezing the tip of condom does not increase risk of condom breakage.

❑ Female condoms

Note: If latex female condoms are available in the setting where the tools will be used, indicate that oil-based lubricants should not be used with latex female condoms. However, for female condoms made from nitrile or polyurethane, any lubricant, including oil-based, is acceptable.

❑ Fertility awareness methods/Standard Days Method

❑ Lactational Amenorrhea Method (LAM)

❑ Spermicides

❑ Diaphragms

❑ Withdrawal

12. Side effect management

Information about management of side effects was omitted from all digital tools included in the content review, yet these tools are optimally designed to support self-care, including after-care instructions and reassurance regarding common side effects.

Verify the presence of information about side effect management for the below methods, as users of the following methods may experience side effects.

Include basic information on use of NSAIDs (Aspirin, Ibuprofen, or Naproxen) to alleviate irregular bleeding with hormonal methods other than COCs, or in case of menstrual cramps with IUD. Access to menstrual products can help to manage irregular and/or prolonged bleeding that commonly occurs with some of the methods.

- ❑ Combined oral contraceptive pill (COCs)
- ❑ Progestin-only pills (POPs)
- ❑ Emergency contraceptive pills (ECPs)
- ❑ Injectable(s)
- ❑ Combined patch
- ❑ Combined vaginal ring
- ❑ Implant(s)
- ❑ Copper-Bearing Intrauterine Device (Cu-IUD, IUCD)
- ❑ Levonorgestrel Intrauterine Device (hormonal IUD)
- ❑ Tubal ligation
- ❑ Vasectomy

TIPS

Many women seek information about the locations where each method can be accessed and any limiting factors. It is helpful to share which methods require a clinic visit and which do not.

13. Where to obtain methods

Ensure it is clear whether the tool is offering a link for an online consultation versus information about where a method can be accessed.

Verify that the tool:

- ❑ instructs users how to obtain each method,
- ❑ shares the location of a nearby provider, health facility, pharmacy, or online ordering system where the method(s) can be obtained, or
- ❑ offers a referral to a nearby health facility.

TABLE
CONTENT AREAS BY WEIGHT

The content areas with a weight of 1 are essential for high quality, comprehensive digital family planning tools. There may be some tools which by design do not integrate information about those content areas with a weight less than 1. For any content areas excluded from your tool, carefully consider the justification and include it below.

SAMPLE JUSTIFICATION: Our tool did not address "mechanism of action" because this family planning tool is designed to generate demand, which it does by sharing basic information via text message. Messages must be within the character count for a standard SMS message and content is limited to key messages designed to generate interest and encourage users to seek additional information from another source to make an informed decision. We determined that mechanism of action is not a key message for demand generation.

Content Area	Weight	Justification for exclusion, if applicable
Side effect management	.3	
Reproductive intentions	.6	
Mechanism of action	.6	
Discreetness	.7	
Non-contraceptive benefits	.7	
Return to fertility	1	
Instructions for use	1	
Dual protection	1	
Duration of protection	1	
Side effects	1	
Method effectiveness	1	
Names all modern methods	1	
